# Artificial intelligence for ultrasound-based differentiation of soft tissue liposarcoma from benign masses

**DOI:** 10.3389/fmed.2026.1960900

**Published:** 2026-10-07

**Authors:** YuKun Deng, Chengjia Qu, Yi Cao, Guoyang Yu, Jingmiao Yu, Jie Yang, Wen Guo, Dandan Wang, Xiaoting Qin, Yibing Zhao, Tao Chen

**Affiliations:** 1 Department of Medical Ultrasonics, Beijing Jishuitan Hospital Affiliated to Capital Medical University, National Center for Orthopedics, Beijing, China; 2 Department of Vascular Surgery, Beijing Jishuitan Hospital Affiliated to Capital Medical University, National Center for Orthopedics, Beijing, China

**Keywords:** artificial intelligence, liposarcoma, ResNet50, ultrasound, u-net

## Abstract

**Background:**

Accurate preoperative differentiation of liposarcoma from benign masses on ultrasound remains challenging and operator-dependent. We aimed to develop and validate a deep learning-based model to assist in distinguishing liposarcoma from benign masses.

**Methods:**

In this retrospective single-center study, we collected B-mode ultrasound examinations from patients with soft tissue masses between March 2015 and March 2022 at Beijing Jishuitan Hospital affiliated to Capital Medical University. A two-stage deep convolutional neural network was developed: a U-Net model segmented tumor regions, followed by a ResNet50 classifier for tumor type prediction. The cohort (*n* = 317) was split at the patient level into a development set (*n* = 222) and an internal test set (*n* = 95). Model performance was compared with 4 radiologists in the internal test set.

**Results:**

Among 317 cases, 191 were benign masses and 126 were liposarcomas. In the internal test set, the AI model achieved an AUC of 0.97 (95% CI 0.92–1.00), an accuracy of 0.97 (95% CI 0.93–1.00), a sensitivity of 0.96 (95% CI 0.86–1.00), and a specificity of 0.97 (95% CI 0.93–1.00). AI achieved significantly higher accuracy than both residents after Holm adjustment (adjusted *P* = 0.0156 and 0.0352, respectively). Differences between AI and either specialist were not statistically significant (both adjusted *P* = 1.0000).

**Conclusions:**

A two-stage deep learning model using ultrasound images showed preliminary potential for differentiating liposarcoma from benign masses in this single-center cohort. External validation is required before clinical use.

## Introduction

Soft tissue sarcoma is a rare malignant tumor, accounting for 1% of adult solid malignant tumors (1). Liposarcoma is the second most common form of malignant soft-tissue tumor, accounting for 10%–35% of such cases (2). Liposarcoma is mainly derived from primitive mesenchymal cells and usually occurs in the lower limbs and retroperitoneum of the human body. It has intermediate malignant biological characteristics (3). In current practice, diagnosis relies on a combination of clinical evaluation, imaging, and histopathology. Cross-sectional imaging such as MRI and CT can provide valuable anatomic and tissue information, but these modalities may be limited by cost, availability, and contrast-agent requirements (4). Doppler ultrasound, in contrast, is non-invasive, widely accessible, and cost-effective, making it an attractive first-line tool for evaluating superficial and limb soft-tissue masses (5, 6). However, ultrasound interpretation remains highly operator-dependent, and diagnostic accuracy can vary considerably—particularly among less experienced readers—contributing to misclassification of liposarcoma as benign entities (e.g., lipoma or fibroma) and potentially delaying optimal treatment.

Recent advances in artificial intelligence (AI), especially deep learning (DL), offer an opportunity to improve diagnostic consistency in ultrasound (7, 8). Deep convolutional neural networks (DCNNs) have demonstrated strong performance in automated feature extraction and image classification, supporting the development of computer-aided diagnosis systems that can assist clinicians by providing objective, reproducible predictions (9, 10). Many prior ultrasound DCNN studies have focused on benign/malignant differentiation in organ-based applications (11–18), while recent studies have also extended deep learning approaches to the ultrasound evaluation of soft-tissue tumors (19).

This study investigated a two-stage U-Net/ResNet50 framework for differentiating liposarcoma from benign soft-tissue masses on ultrasound. The evaluation was organized around the contributions of lesion localization and classifier architecture. Whole-image and ROI-based comparisons address the role of localization; frozen-feature classifiers and sonographic descriptor-based models provide simpler reference models; and alternative backbones address the influence of architecture choice. The objective was to assess the framework's potential for this diagnostic task and clarify the evidence needed for further validation.

## Methods

### Study design and participants

The overall workflow of the AI model is illustrated in the Central Illustration. As shown in Figure 1, 504 patients between March 2015 and March 2022 in our hospital were enrolled in this study. 185 patients with incomplete baseline information were excluded. Furthermore, two patients with atypical lipomatous tumors were excluded because this entity is generally considered a borderline or intermediate lipomatous tumor and may overlap with both benign lipomatous lesions and well-differentiated liposarcoma. In the end, a total of 317 cases were included in the final analysis, comprising 191 benign masses and 126 liposarcomas. The mean age of the whole cohort was 51.6 years, with 178 male and 139 female patients. The baseline characteristics of these patients grouped by benign masses and liposarcoma are shown in Table 1. The detailed pathological types of 191 benign masses are presented in Supplement Table S2. To further describe the heterogeneity of the benign group, we summarized clinical and ultrasound characteristics by benign pathological category in Supplement Table S3.

**Central Illustration:**
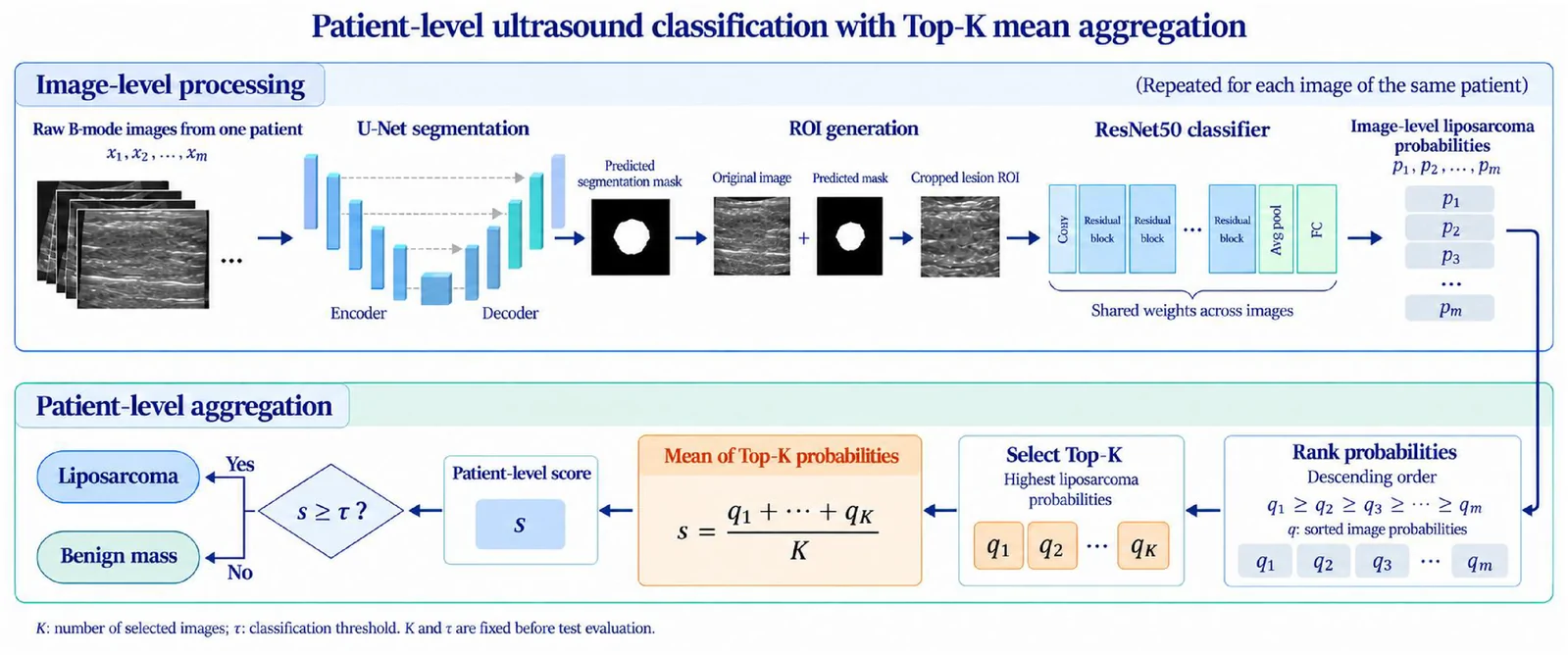
Patient-level ultrasound classification. U-Net localized lesion regions for ResNet50 classification. Image-level liposarcoma probabilities were ranked within each patient, and the highest K probabilities were averaged to obtain a patient-level score.

**Figure 1:**
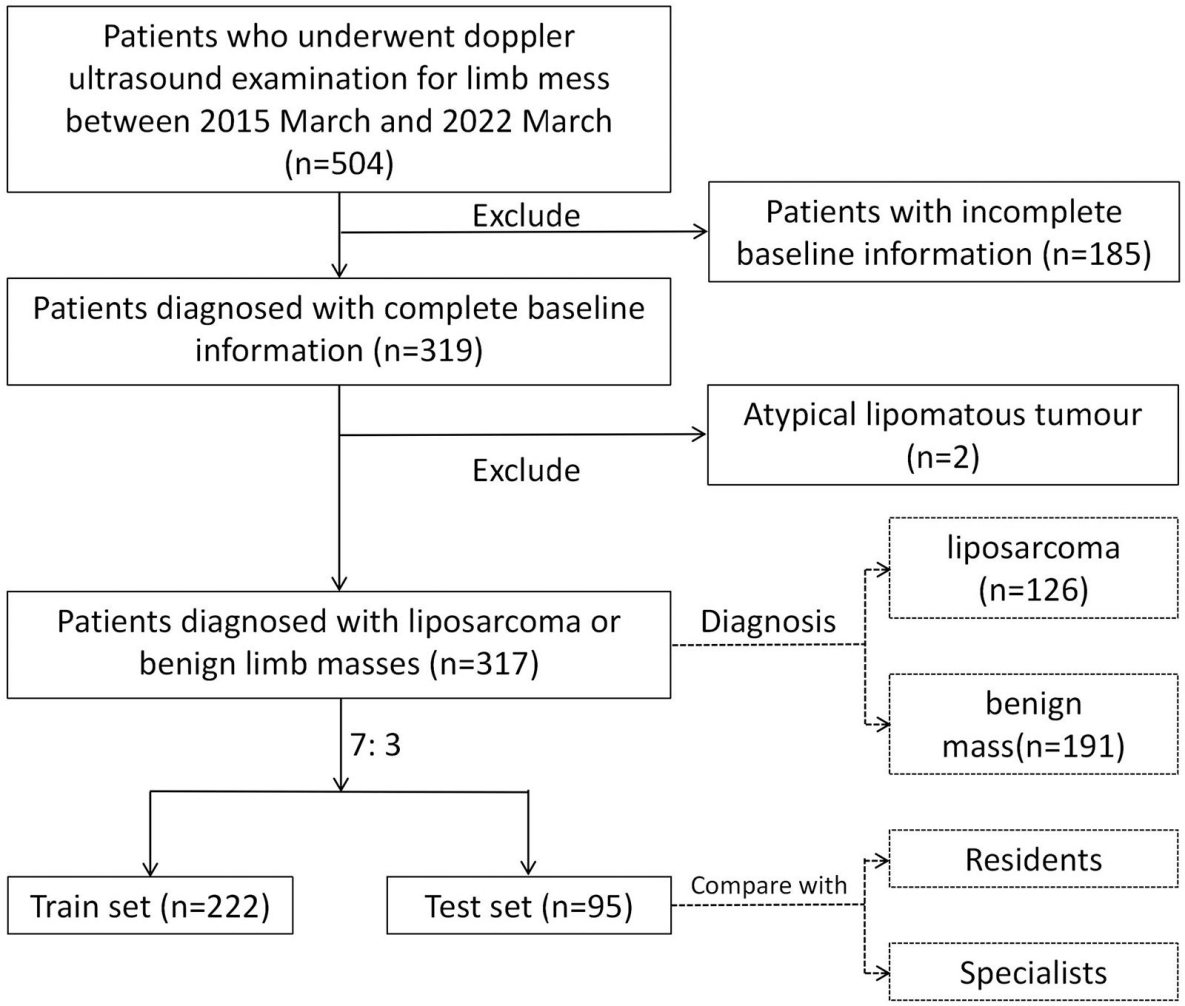
Study flow chart.

**Table 1:** Clinical characteristics of cohort.

Patients' characteristics	Total (*n* = 317)	Benign masses (*n* = 191)	Liposarcoma (*n* = 126)	*p* value
Age (years)	51.6 ± 14.34	48 ± 9.99	55.6 ± 13.25	<0.001
Male [% (*n*)]	178 (56.15)	105 (54.97)	73 (57.9)	0.644
tumor size (cm)	4.40 (2.48, 9.60)	3.10 (1.90, 4.60)	11.10 (7.50, 15.80)	<0.001
Tumor site				<0.001
Head/neck [% (*n*)]	34 (10.73)	33 (17.28)	1 (0.79)	
Trunk [%(n)]	110 (34.70)	94 (49.21)	16 (12.70)	
Upper limb [% (*n*)]	45 (14.20)	36 (18.85)	9 (7.14)	
Lower limb [% (*n*)]	128 (40.38)	28 (14.66)	100 (79.37)	
Echogenicity				<0.001
Hypoechoic [% (*n*)]	157 (49.53)	77 (40.31)	80 (63.49)	
Isoechoic [% (*n*)]	33 (10.41)	26 (13.61)	7 (5.56)	
Hyperechoic [% (*n*)]	116 (36.59)	78 (40.84)	38 (30.16)	
Mixed-echogenicity [% (*n*)]	11 (3.47)	10 (5.24)	1 (0.79)	
Adler grade				<0.001
0 [% (*n*)]	112 (35.33)	81 (42.41)	31 (24.60)	
1 [% (*n*)]	99 (31.23)	63 (32.98)	36 (28.57)	
2 [% (*n*)]	29 (9.15)	13 (6.81)	16 (12.70)	
3 [% (*n*)]	77 (24.29)	34 (17.80)	43 (34.13)	
Homogeneity [% (*n*)]	79 (24.92)	76 (39.79)	3 (2.38)	<0.001
Regular [% (*n*)]	251 (79.18)	191 (100.00)	60 (47.62)	<0.001
Circumscribed [% (*n*)]	195 (61.71)	115 (60.21)	80 (64.00)	0.498
Linear echogenic strands [% (*n*)]	196 (61.83)	116 (60.73)	80 (63.49)	0.621
Anechoic area and calcification [% (*n*)]	47 (14.83)	19 (9.95)	28 (22.22)	0.003
Pain [% (*n*)]	10 (3.15)	1 (0.52)	9 (7.14)	0.003

### Train-test split

The dataset was randomly divided at the patient level into a development set and an internal test set at a ratio of 7:3. The development set included 222 patients, and the test set included 95 patients. Epoch selection, checkpoint selection, hyperparameter tuning, and threshold selection were restricted to the development set; the internal test set was excluded from all model-development decisions and used only for final evaluation after the model and evaluation settings had been fixed. The distribution of baseline characteristics between the two sets is shown in Supplement Table S1. Both sets were drawn from the same single-center cohort.

### Ultrasonography acquisition

Ultrasound examinations were performed using a Philips iU22 ultrasound system with standard manufacturer-provided probes. A high-frequency linear-array probe L12–5 was used for superficial lesions, with a B-mode frequency range of 5.0−12.0 MHz, Doppler frequency range of 7.5 MHz, and imaging depth of approximately 3–8 cm. For large or deep lesions, a C5-2 convex-array probe was used, with a B-mode frequency range of 2–5 MHz, Doppler frequency range of 3.5 MHz, and imaging depth of approximately 8–20 cm. Lesions and adjacent tissues were scanned in multiple planes. B-mode ultrasound was used to assess lesion location, size, shape, boundary, echogenicity, internal echo pattern, and relationship with adjacent structures. Color Doppler ultrasound was used to evaluate internal vascularity according to the Adler semiquantitative grading system (20). The ultrasound images of liposarcoma and benign masses are shown in Figure 2. Panel (a) shows a liposarcoma, panel (b) a lipoma, panel (c) a vascular lesion, panel (d) a cystic lesion, panel (e) a fibrous lesion, and panel (f) a peripheral nerve lesion. B-mode and available color Doppler images are shown.

**Figure 2:**
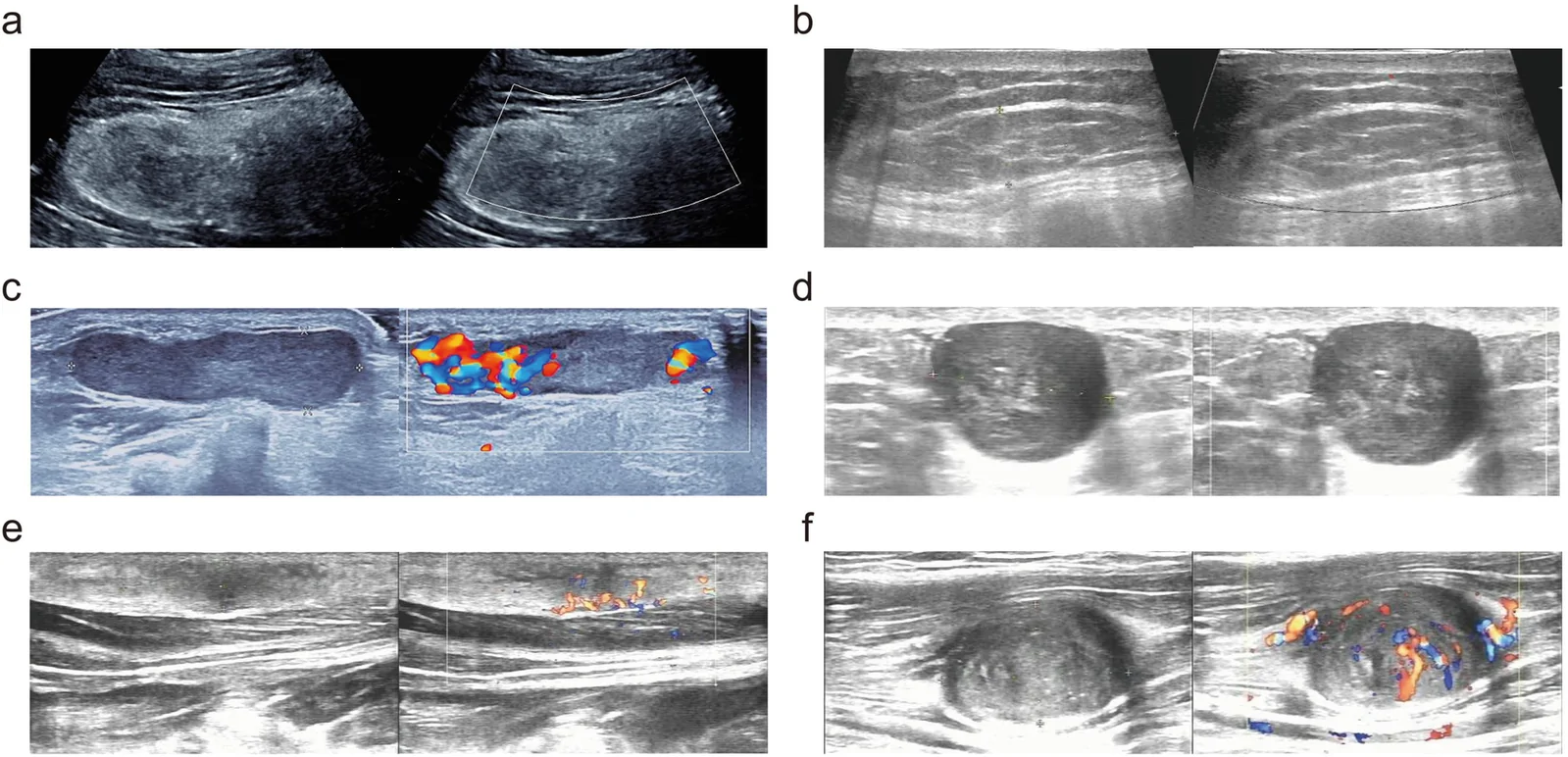
Representative ultrasound images of liposarcoma and benign masses: **(a)** liposarcoma; **(b)** lipoma; **(c)** vascular lesion; **(d)** cystic lesion; **(e)** fibrous lesion; and **(f)** peripheral nerve lesion. B-mode images are shown for all lesions, with corresponding color Doppler images where available.

Ultrasound examinations were stored in DICOM format. Videos were converted into consecutive frames using MicroDicom DICOM Viewer version 2.8.3 (MicroDicom Ltd., Sofia, Bulgaria). Clinical and imaging data were retrospectively collected from the hospital electronic medical record system and picture archiving and communication system. The collected variables included age, sex, symptoms, history of malignancy, lesion location, lesion size, ultrasound features, Doppler blood-flow grade, pathological diagnosis, and ultrasound images/videos.

### DCNN-based localization and classification framework

We developed a two-stage DCNN framework consisting of a U-Net lesion localization network and a ResNet50 classification network. U-Net identified the lesion region in each ultrasound image (21), and the resulting region of interest (ROI) was passed to ResNet50 to distinguish liposarcoma from benign masses. Image-level predictions were subsequently combined using the patient-level aggregation procedure described below. The overall framework is shown in the Central Illustration.

The U-Net network was trained using radiologist-annotated lesion masks as ground truth. Its encoder extracted image features at multiple scales, while its decoder combined these features to delineate the lesion boundary. The predicted mask was used to extract the lesion ROI for subsequent classification. This stage was intended to reduce the influence of surrounding tissues and background information, allowing the classifier to focus on the ultrasound appearance of the mass.

The ResNet50 backbone was pretrained on ImageNet and fine-tuned using the lesion ROIs. It extracted imaging features from each ROI and generated a prediction probability for liposarcoma. Thus, U-Net provided lesion localization, whereas ResNet50 performed diagnostic classification.

### Comparison models and ablation configurations

Ten comparator models were included to assess alternative classification strategies, lesion localization approaches, classification backbones, and sonographic descriptor-based models.

Three models used features extracted from U-Net-derived ROIs by a frozen, ImageNet-pretrained ResNet50: (1) L1-regularized logistic regression, providing a regularized linear classification baseline; (2) support vector machine (SVM), providing an alternative conventional classifier; and (3) random forest, providing a tree-based classification baseline. These models assessed whether frozen image representations combined with conventional classifiers could provide competitive performance without fine-tuning ResNet50.

Two models examined the contribution of lesion localization: (4) whole-image ResNet50, which removed U-Net and classified complete ultrasound images; and (5) manual-ROI ResNet50, which replaced U-Net-derived ROIs with manually delineated ROIs while retaining ResNet50 for classification. The manual ROIs were delineated by an expert radiologist who was not involved in model evaluation. These comparisons assessed the value of lesion localization and the effect of automatic vs. manual ROI extraction.

Two models examined the choice of classification backbone: (6) U-Net/MobileNetV3-Small, which retained U-Net localization and replaced ResNet50 with a lightweight convolutional neural network; and (7) U-Net/Swin-T, which retained U-Net localization and used a pretrained Swin Transformer Tiny, a hierarchical vision Transformer, for classification.

Three additional models used the same recorded sonographic descriptors as predictors: (8) L1-regularized logistic regression, (9) SVM, and (10) random forest. These models assessed whether conventional ultrasound characteristics could distinguish liposarcoma from benign masses without deep image feature extraction.

### Image preprocessing, model development and validation

All ultrasound videos were converted into consecutive image frames with a sampling interval of 1s, and frames with poor image quality, severe acoustic artifacts, or incomplete lesion visualization were excluded before model development by radiologists blinded to the pathological diagnosis. Each patient contributed approximately 6–7 frames in the benign cohort and 12–13 frames in the liposarcoma cohort. All images were resized to 572 × 572 pixels and normalized to [0, 1] before network input. A total of 2,964 ultrasound images from 317 patients were retained. Of these, 2,176 images from 222 patients with radiologist-annotated lesion masks as the ground truth were assigned to the development set, and 788 images from 95 patients were assigned to the internal test set. The dataset was split at the patient level to ensure that images from the same patient did not appear in both sets. The U-Net model was trained for 100 epochs with a batch size of 8 using the Adam optimizer with an initial learning rate of 0.0001, and the model with the best validation performance was selected for lesion localization. The lesion regions identified by U-Net were subsequently used as inputs for downstream classification. A ResNet50 classifier pretrained on ImageNet was fine-tuned to distinguish liposarcoma from benign masses and trained for 50 epochs with a batch size of 16 using stochastic gradient descent with a momentum of 0.9 and a weight decay of 0.0005. The reference diagnosis for each patient was liposarcoma or benign mass, as recorded in the medical records. The same image preprocessing was applied to the internal test set. Segmentation results were assessed by the Dice coefficient, intersection over union (IoU), and localization accuracy.

For patient-level prediction, ResNet50 generated a liposarcoma probability for each ultrasound image. Image-level probabilities were aggregated using a top-K mean strategy. Specifically, the probabilities for each patient were ranked in descending order, and the K highest probabilities were averaged to obtain a single patient-level prediction score. For patients with fewer than K images, all available probabilities were averaged. The cutoff value for liposarcoma classification was determined by K-means clustering using patient-level prediction score. Patient-level stratified five-fold cross-validation was performed within the development set, with stratification according to diagnosis (liposarcoma vs. benign mass). In each fold, approximately 177–178 patients were used for training and 44–45 for validation. Both U-Net and ResNet50 were trained separately within each fold.

Model performance was evaluated in the internal test set and compared with that of four radiologists: two residents with 2 and 3 years of experience and two specialists with 10 and 13 years of experience. All readers independently reviewed the B-mode ultrasound images grouped by patient, with cases presented in random order, and provided one diagnosis per patient. They were blinded to the final diagnosis and did not participate in data preparation. Both the radiologists and the AI model were evaluated on the same patients in the internal test set. We also performed subgroup analyses to evaluate the model's robustness.

### Statistical methods

Model development and statistical analyses were performed using R (version 4.4.2) and Python (version 3.10.10). Categorical variables are presented as numbers (%) and were analyzed using Pearson's chi-square test or Fisher's exact test. Continuous variables are presented as mean ± standard deviation (mean ± SD) for normally distributed data or median (interquartile range) (M[Q1-Q3]) for non-normally distributed data. Group comparisons were performed using Student's *t*-test (normal distribution) or the Mann–Whitney *U*-test (non-normal distribution). Diagnostic performance was evaluated at the patient level, with liposarcoma defined as the positive class. ROC curves and AUC values were calculated for all models from continuous patient-level prediction scores using the pROC package. Accuracy, sensitivity, specificity, positive predictive value (PPV), and negative predictive value (NPV) were used to evaluate both the models and the radiologists. Each prediction was classified as correct or incorrect relative to the reference diagnosis. Differences in accuracy between AI and each reader were assessed using two-sided exact McNemar tests. Results with *P*-values of less than 0.05 were considered to indicate a statistically significant difference.

## Results

### Demographic data and lesion characteristics

Patients with liposarcoma were older and had markedly larger tumors than patients with benign masses. Sex distribution did not differ significantly between groups. tumor location differed significantly: liposarcomas were predominantly located in the lower limb, whereas benign lesions were more frequently observed in the trunk and head/neck. On ultrasound, echogenicity patterns differed between groups, with liposarcomas more often hypoechoic. Homogeneity and lesion shape regularity also differed: homogeneous appearance was uncommon in liposarcoma, and regular morphology was less frequent in liposarcoma. Anechoic areas/calcification were more common in liposarcoma. Doppler Adler grade differed significantly, with a higher proportion of grade 3 flow in liposarcoma. Pain was reported more frequently in liposarcoma (Table 1).

Among the 191 benign masses, pathological diagnoses included lipoma (*n* = 96), hemangioma or other vascular lesions (*n* = 33), epidermoid/sebaceous cysts (*n* = 18), fibroma/fibromatosis-related lesions (*n* = 7), peripheral nerve lesions (*n* = 2), and other benign lesions (*n* = 35). These pathological categories reflected the heterogeneity of benign soft-tissue masses included in this study (Supplement Table S2). Lipomas were most commonly located in the trunk and were mainly hyperechoic, with linear echogenic strands observed in all cases. Vascular lesions were more frequently located in the extremities, often presented as hypoechoic or mixed-echogenicity lesions, and showed higher Adler Doppler grades. Cystic lesions were predominantly hypoechoic and more commonly showed anechoic areas or calcification. Fibrous lesions were relatively larger, frequently located in the trunk, and mainly showed hyperechoic patterns with linear echogenic strands. Peripheral nerve lesions were all located in the upper limb and showed hypoechoic features (Supplement Table S3).

### Patient-level aggregation and classification threshold

For patient-level aggregation, the top-K parameter was set to *K* = 3. The classification cutoff was determined by K-means clustering within the development cohort, with a final threshold of 0.506. These settings were fixed before evaluation in the 95-patient internal test set.

### Lesion localization performance

The U-Net model achieved a Dice coefficient of 0.86, IoU of 0.76, and localization accuracy of 0.91 in the test set, suggesting that the lesion regions could be reasonably localized before ResNet50-based classification.

### Comparison with baseline models and ablation configurations

Figure 3 shows the AUCs of baseline and ablation models. In the 95-patient internal test set, U-Net/ResNet50 achieved an AUC of 0.97 (95% CI 0.92–1.00). Among the models using frozen ResNet50 features, L1-regularized logistic regression, SVM, and random forest achieved AUCs of 0.93 (95% CI 0.87–0.98), 0.96 (95% CI 0.93–0.99), and 0.96 (95% CI 0.91–0.99), respectively. For the localization comparisons, whole-image ResNet50 achieved an AUC of 0.86 (95% CI 0.77–0.93), whereas ResNet50 using manual ROIs achieved an AUC of 0.84 (95% CI 0.75–0.92). For the classification backbone comparisons, U-Net-MobileNetV3-Small and U-Net-Swin-T achieved AUCs of 0.82 (95% CI 0.71–0.91) and 0.88 (95% CI 0.80–0.95), respectively. The sonographic descriptor-based models achieved AUCs of 0.52 (95% CI 0.38–0.67) for L1-regularized logistic regression, 0.64 (95% CI 0.51–0.77) for SVM, and 0.65 (95% CI 0.53–0.77) for random forest. All confidence intervals were calculated using 2,000 case-level percentile bootstrap resamples.

**Figure 3:**
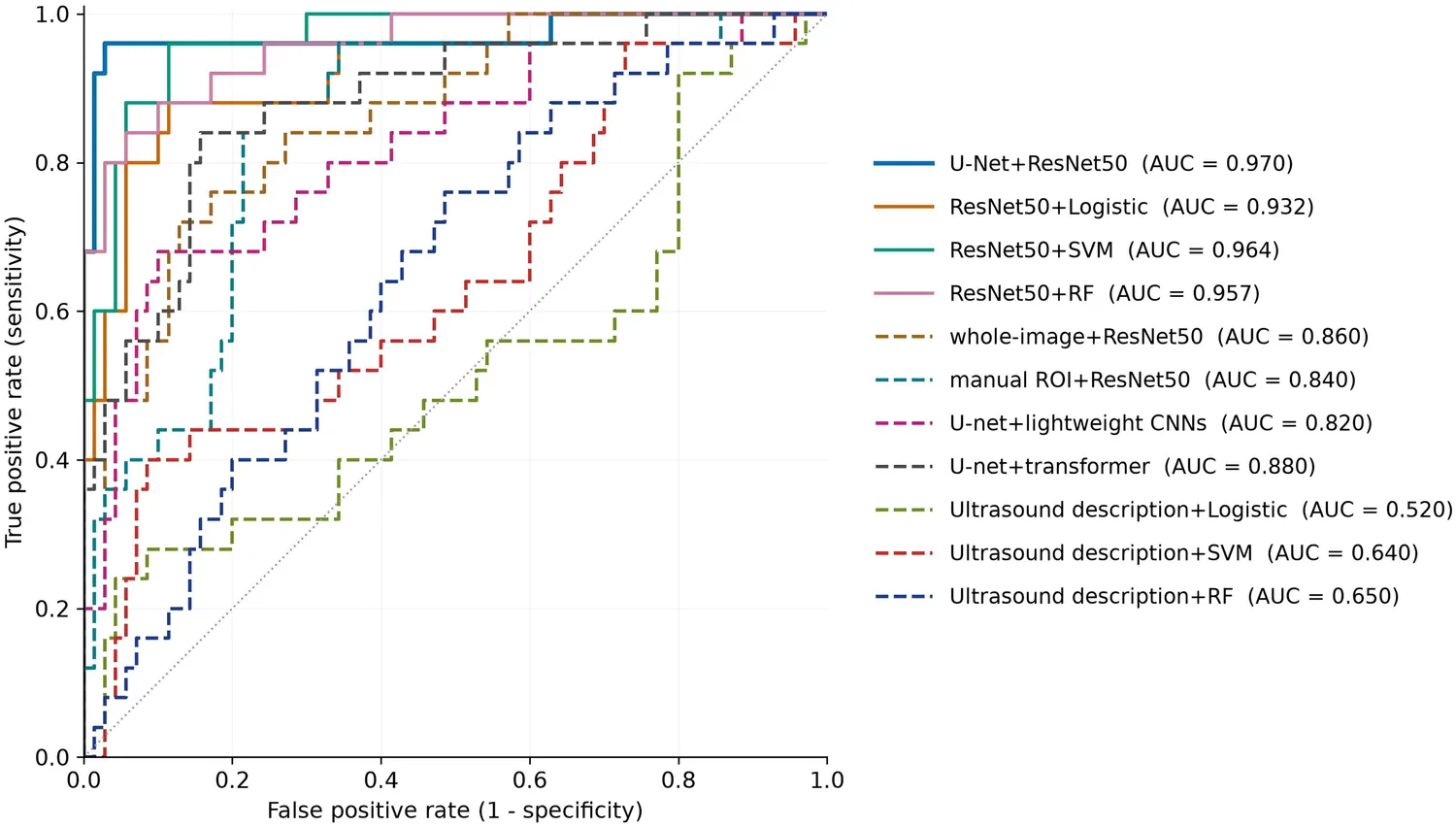
Receiver operating characteristic (ROC) curves of the U-net/ResNet50 model and ten comparator models in the 95-patient internal test set. AUC, area under the ROC curve; ROI, region of interest; SVM, support vector machine; RF, random forest.

### Performance of the artificial intelligence model vs. radiologists

As shown in Table 2, in the internal test set, the AI model achieved an AUC of 0.97 (95% CI 0.92–1.00), an accuracy of 0.97 (95% CI 0.93–1.00), a sensitivity of 0.96 (95% CI 0.86–1.00), and a specificity of 0.97 (95% CI 0.93–1.00). The PPV was 0.92 (95% CI 0.81–1.00), and the NPV was 0.99 (95% CI 0.95–1.00). The two specialist readers achieved accuracies of 0.95 (95% CI 0.89–0.99) and 0.95 (95% CI 0.91–0.99), sensitivities of 0.80 (95% CI 0.63–0.95) and 0.84 (95% CI 0.69–0.96), and specificities of 1.00 (95% CI 1.00–1.00) and 0.99 (95% CI 0.95–1.00), respectively. Their mean accuracy, sensitivity, and specificity were 0.95, 0.82, and 1.00, respectively. The two resident readers achieved accuracies of 0.87 (95% CI 0.80–0.94) and 0.87 (95% CI 0.80–0.94), sensitivities of 0.68 (95% CI 0.50–0.85) and 0.72 (95% CI 0.54–0.89), and specificities of 0.94 (95% CI 0.88–0.99) and 0.93 (95% CI 0.86–0.99), respectively. Their mean accuracy, sensitivity, and specificity were 0.87, 0.70, and 0.94, respectively. The confusion matrices for the 95-patient internal test set, comprising 25 liposarcoma cases and 70 benign masses, are shown in Figure 4. The AI model correctly classified 24 liposarcoma cases and 68 benign masses, with one false negative and two false positives. Specialist readers 1 and 2 correctly identified 20 and 21 liposarcoma cases and 70 and 69 benign masses, respectively, resulting in five and four false negatives and zero and one false positive. Resident readers 1 and 2 correctly identified 17 and 18 liposarcoma cases and 66 and 65 benign masses, respectively, resulting in eight and seven false negatives and four and five false positives. As shown in Table 3, AI achieved significantly higher accuracy than both residents after Holm adjustment (adjusted *P* = 0.016 and 0.035, respectively). Differences between AI and either specialist were not statistically significant (both adjusted *P* = 1.000). Subgroup analyses are presented in Supplement Table S4.

**Table 2:** Performance of the AI model and participating radiologists.

AI model / Radiologist	Accuracy (95%CI)	Sensitivity (95%CI)	Specificity (95%CI)	PPV (95%CI)	NPV (95%CI)
U-Net/ResNet50	0.97 (0.93–1.00)	0.96 (0.86–1.00)	0.97 (0.93–1.00)	0.92 (0.81–1.00)	0.99 (0.95–1.00)
Specialist1	0.95 (0.89–0.99)	0.80 (0.63–0.95)	1.00 (1.00–1.00)	1.00 (1.00–1.00)	0.93 (0.87–0.99)
Resident1	0.87 (0.80–0.94)	0.68 (0.50–0.85)	0.94 (0.88–0.99)	0.81 (0.62–0.95)	0.89 (0.82–0.96)
Specialist2	0.95 (0.91–0.99)	0.84 (0.69–0.96)	0.99 (0.95–1.00)	0.95 (0.84–1.00)	0.95 (0.89–0.99)
Resident2	0.87 (0.80–0.94)	0.72 (0.54–0.89)	0.93 (0.86–0.99)	0.78 (0.61–0.94)	0.90 (0.83–0.96)

Bold indicates statistical significance (p < 0.05).

**Figure 4:**
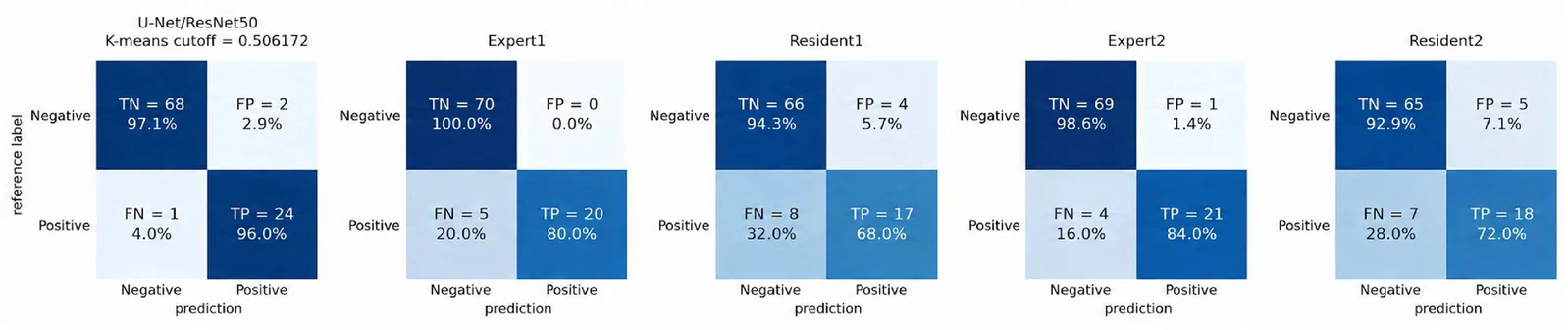
Confusion matrices for the AI model and participating readers, with liposarcoma as the positive class.

**Table 3:** Paired comparisons of diagnostic accuracy between AI and individual readers.

Reader	AI accuracy, n/N (%)	Reader accuracy, n/N (%)	Both correct, n	Both incorrect, n	AI correct/reader incorrect, n	AI incorrect/reader correct, n	Exact McNemar P	Holm-adjusted P
Resident 1	92/95 (96.8)	83/95 (87.4)	83	3	9	0	0.0039	**0.016**
Resident 2	92/95 (96.8)	83/95 (87.4)	82	2	10	1	0.0117	**0.035**
Specialist 1	92/95 (96.8)	90/95 (94.7)	89	2	3	1	0.6250	1.000
Specialist 2	92/95 (96.8)	90/95 (94.7)	88	1	4	2	0.6875	1.000

### Web-based implementation

We established the LipoScope platform based on U-Net/ResNet50 framework (Figure 5, https://liposcope.com). The platform supports uploading multiple ultrasound images from a single case, displaying the predicted region of interest, and reporting image-based classification results. A case-level malignancy score is obtained by averaging the three highest image-level scores, or all available scores when fewer than three images are uploaded. The interface also displays the number of images analyzed and inference time.

**Figure 5:**
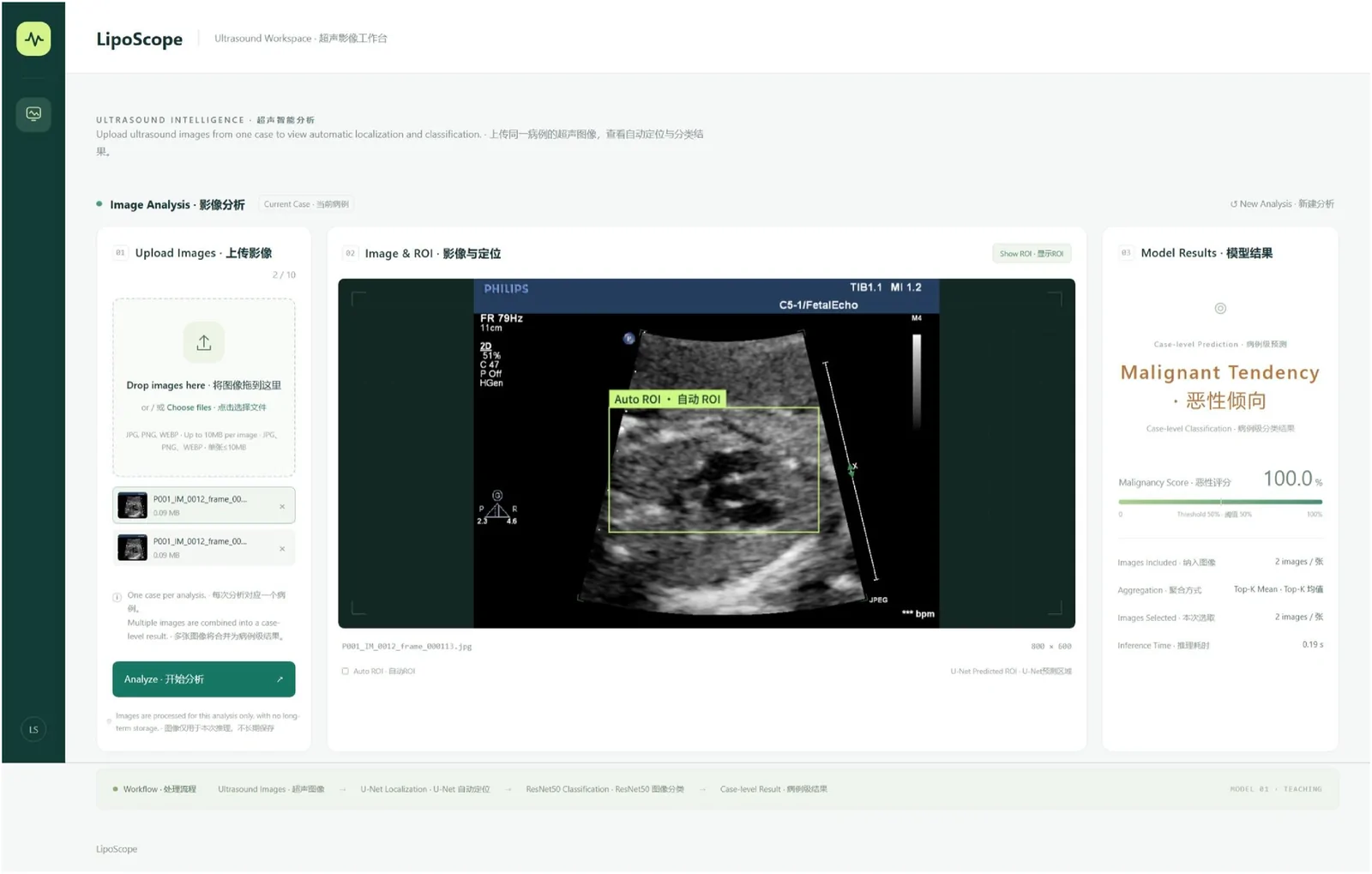
Web interface of the lipoScope ultrasound image analysis platform. Users upload ultrasound images from a single case (left). U-Net predicts a region of interest, displayed as a bounding box, which is subsequently classified by ResNet50 (center). The platform reports the case-level classification and malignancy score, calculated by averaging the highest three image-level scores, or all available scores when fewer than three images are uploaded (right).

## Discussion

In this study, we developed and internally evaluated a two-stage deep learning framework combining U-Net-based lesion localization with ResNet50-based classification to differentiate liposarcoma from benign masses on ultrasound. As a widely available, cost-effective, and non-invasive modality, ultrasound provides a practical setting for investigating AI-assisted lesion assessment. Previous studies have explored AI for tumor characterization across imaging modalities (22–24) and for ultrasound-based diagnosis of superficial soft-tissue masses (19, 25). Our findings provide preliminary proof-of-concept evidence for this specific diagnostic task in a retrospective single-center cohort. Further studies are needed to establish external generalizability and determine whether model-assisted assessment improves diagnostic decisions or patient outcomes.

Wang et al. evaluated a framework combining image-based deep learning and language-model assistance for ovarian tumor diagnosis and reported improvements in participating readers' diagnostic performance (26). This work supports investigating AI-assisted ultrasound assessment. For image-based classification, an important design question is whether the network should process the whole image or an explicitly localized lesion. Whole-image classification preserves surrounding tissue information but may also allow background structures and acquisition-related features to influence predictions. Lesion localization offers a way to constrain the classifier's input, although its value depends on localization accuracy and the information retained around the lesion. U-Net was developed for biomedical image segmentation, combining contextual information with spatial detail to support localization. ResNet was developed to facilitate the optimization of deeper convolutional networks through residual learning. These complementary functions provide a rationale for combining U-Net-based localization with ResNet50-based classification.

The limited availability of independent patients also makes the choice of classification strategy important. Fine-tuning a pretrained network allows its representations to adapt to the target task, whereas frozen image features combined with conventional classifiers reduce the number of network parameters estimated from the study cohort. Karacor and Sahin investigated a data-efficient approach integrating image-derived features with sonographic descriptors for breast ultrasound lesion classification (27). Their study provides a rationale for considering image-feature and descriptor-based approaches alongside deep-network fine-tuning. For the present task, such comparisons can help determine whether additional model complexity provides useful discrimination.

Classifier comparisons address a related question: how much task-specific model adaptation is necessary in a relatively small cohort? Classifiers trained on features extracted by a frozen pretrained ResNet50 were included to assess whether simpler alternatives to the fine-tuned network could effectively distinguish liposarcoma from benign masses, while a lightweight backbone tests whether a smaller model is sufficient. Sonographic descriptor-based models help assess the added value of learned image features beyond conventional ultrasound characteristics. Sonographic descriptors provide useful diagnostic information but may be insufficient to achieve adequate predictive performance. Analyses stratified by lesion size, location, and pathological subtype further clarified the model's robustness.

## Conclusions

In conclusion, the two-stage model showed preliminary potential for differentiating liposarcoma from benign masses in an internal test set from a single-center cohort. Its performance for other malignant soft-tissue tumors and atypical lipomatous tumors remains unestablished. Larger multicenter, multi-device, and prospective validation studies are required before clinical implementation.

## Limitations

Several limitations should be acknowledged. First, this was a single-center study conducted at a national orthopedic referral center. Patients with suspected malignant bone or soft-tissue tumors are frequently referred to Beijing Jishuitan Hospital from other institutions, and liposarcoma cases were enriched in this cohort. Therefore, the proportion of liposarcoma cases in this study should not be interpreted as the actual prevalence of liposarcoma among all patients with superficial soft-tissue masses, and referral bias may limit the generalizability of the model to general hospitals or primary-care settings. Second, all ultrasound examinations were performed using a single Philips iU22 ultrasound system. Although this reduced acquisition heterogeneity during model development, it may also limit the applicability of the model to images obtained from other ultrasound vendors, newer scanners, or different imaging protocols. Future studies should include multicenter and multi-vendor datasets to evaluate model robustness and determine whether transfer learning or model recalibration is needed. Third, the present model was designed to differentiate liposarcoma from benign masses and did not include other malignant soft-tissue tumors or atypical lipomatous tumors. Therefore, it should not be interpreted as a comprehensive diagnostic model for all soft-tissue malignancies. In particular, the available evidence does not establish whether excluded malignancies would be misclassified as benign. Fourth, 2,964 images represented only 317 independent patients, and consecutive frames from the same patient were correlated. Although patient-level five-fold cross-validation was performed within the development cohort, final performance was estimated using a single 95-patient internal test set. Therefore, the estimates remain vulnerable to partition-specific variation and overfitting. A few misclassified patients in the 95-patient test set could materially change the diagnostic metrics, and resampling cannot compensate for a small test sample. Fifth, the limited reader comparison does not provide sufficient evidence to support clinical deployment. Therefore, the present model is best viewed as a proof-of-concept prototype. Finally, the number of available frames differed between the benign and liposarcoma groups, which may reflect differences in image acquisition and could introduce acquisition-related bias. Future work must include external validation on diverse, multicenter, and multi-vendor datasets before clinical implementation can be considered.

## Data Availability

The data supporting the findings of this study are available from the corresponding author upon reasonable request.
