# Supplementary material for: Artificial intelligence for ultrasound-based differentiation of soft tissue liposarcoma from benign masses
Source: Front Med (Lausanne). 2026 Oct 7;13:1960900. doi: 10.3389/fmed.2026.1960900 (PMC13684072; doi:10.3389/fmed.2026.1960900)
Supplement: Supplementary file 1 [file Table1.docx]

Supplement table 1. Clinical characteristics of development and internal test sets.

|  | **Total (n = 317)** | **Development set (n=222)** | **Internal test set (n = 95)** | **P value** |
| --- | --- | --- | --- | --- |
| **Age (years)** | 51.2 ± 12.4 | 51.4 ± 11.8 | 50.8 ± 13.8 | 0.728 |
| **Male [%(n)]** | 178 (56.15) | 156 (70.27) | 22 (23.16) | <0.001 |
| **Tumor size (cm)** | 4.40 (2.48, 9.60) | 3.10 (1.90, 4.60) | 11.10 (7.50, 15.80) | <0.001 |
| **Tumor site** |  |  |  | 0.203 |
| Head/neck [%(n)] | 34 (10.73) | 25 (11.26) | 9 (9.47) |  |
| Trunk [%(n)] | 110 (34.70) | 70 (31.53) | 40 (42.11) |  |
| Upper limb [%(n)] | 45 (14.20) | 30 (13.51) | 15 (15.79) |  |
| Lower limb [%(n)] | 128 (40.38) | 97 (43.69) | 31 (32.63) |  |
| ****Echogenicity**** |  |  |  | 0.098 |
| Hypoechoic [%(n)] | 157 (49.53) | 111 (50.00) | 46 (48.42) |  |
| **Isoechoic** [%(n)] | 33 (10.41) | 23 (10.36) | 10 (10.53) |  |
| **Hyperechoic** [%(n)] | 116 (36.59) | 84 (37.84) | 32 (33.68) |  |
| **Mixed-echogenicity** [%(n)] | 11 (3.47) | 4 (1.80) | 7 (7.37) |  |
| ****Adler grade**** |  |  |  | 0.629 |
| **0** [%(n)] | 112 (35.33) | 76 (34.23) | 36 (37.89) |  |
| **1** [%(n)] | 99 (31.23) | 72 (32.43) | 27 (28.42) |  |
| **2** [%(n)] | 29 (9.15) | 18 (8.11) | 11 (11.58) |  |
| **3** [%(n)] | 77 (24.29) | 56 (25.23) | 21 (22.11) |  |
| ****Homogeneity** [%(n)]** | 79 (24.92) | 51 (22.97) | 28 (29.47) | 0.220 |
| ****Regular** [%(n)]** | 251 (79.18) | 164 (73.87) | 87 (91.58) | <0.001 |
| ****Circumscribed** [%(n)]** | 195 (61.71) | 129 (58.37) | 66 (69.47) | 0.063 |
| ****Linear echogenic strands** [%(n)]** | 196 (61.83) | 140 (63.06) | 56 (58.95) | 0.490 |
| ****Anechoic area and calcification** [%(n)]** | 47 (14.83) | 36 (16.22) | 11 (11.58) | 0.287 |
| ****Pain** [%(n)]** | 10 (3.15) | 10 (4.50) | 0 (0.00) | 0.080 |

Supplement table 2. Pathological composition of benign masses.

| **Pathological type** | **Number of cases, n** | **Percentage, %** |
| --- | --- | --- |
| Lipoma | 96 | 50.26 |
| Hemangioma | 30 | 15.71 |
| Sebaceous cyst | 14 | 7.33 |
| Fat herniation | 7 | 3.66 |
| Pyogenic granuloma | 6 | 3.14 |
| Elastofibroma | 5 | 2.62 |
| Intramuscular myxoma | 4 | 2.09 |
| Pilomatricoma | 4 | 2.09 |
| Epidermoid cyst | 4 | 2.09 |
| Glomus tumor | 3 | 1.57 |
| Benign myeloma-like lesion | 2 | 1.05 |
| Parotid adenoma | 2 | 1.05 |
| Tenosynovial giant cell tumor | 2 | 1.05 |
| Palmar fibromatosis | 1 | 0.52 |
| Pleomorphic adenoma of the parotid gland | 1 | 0.52 |
| Ulnar nerve schwannoma | 1 | 0.52 |
| Fibromatous hyperplasia | 1 | 0.52 |
| Morton's neuroma | 1 | 0.52 |
| Hematoma with partial organization | 1 | 0.52 |
| Synovial chondromatosis | 1 | 0.52 |
| Fat pad | 1 | 0.52 |
| Synovial osteochondroma | 1 | 0.52 |
| Xanthoma | 1 | 0.52 |
| Organized hematoma | 1 | 0.52 |
| Diagnosis undetermined | 1 | 0.52 |
| Total | 191 | 100.00 |

Supplement table 3. Clinical characteristics of benign masses.

| **Variable** | **Lipoma (n=96)** | **Vascular lesions (n=33)** | **Cystic lesions (n=18)** | **Fibrous lesions (n=7)** | **Peripheral nerve lesions (n=2)** | **Other benign lesions (n=35)** | **P value** |
| --- | --- | --- | --- | --- | --- | --- | --- |
| **Age (years)** | 48.2±10.0 | 42.2±11.1 | 45.4±11.0 | 52.0±8.7 | 46.6±9.8 | 50.0±11.4 | 0.029 |
| **Male [%(n)]** | 53 (55.21) | 17 (51.52) | 9 (50.00) | 4 (57.14) | 1 (50.00) | 21 (60.00) | 0.981 |
| **Tumor size (cm)** | 3.50 (2.38, 5.12) | 2.80 (1.30, 3.70) | 2.80 (2.12, 3.85) | 5.10 (3.30, 7.65) | 3.20 (1.85, 4.55) | 2.50 (1.80, 3.70) | <0.001 |
| **Tumor site** |  |  |  |  |  |  | <0.001 |
| Head/neck [%(n)] | 18 (18.75) | 3 (9.09) | 3 (16.67) | 0 (0.00) | 0 (0.00) | 9 (25.71) |  |
| Trunk [%(n)] | 57 (59.38) | 7 (21.21) | 9 (50.00) | 5 (71.43) | 0 (0.00) | 16 (45.71) |  |
| Upper limb [%(n)] | 10 (10.42) | 13 (39.39) | 5 (27.78) | 1 (14.29) | 2 (100.00) | 5 (14.29) |  |
| Lower limb [%(n)] | 11 (11.46) | 10 (30.30) | 1 (5.56) | 1 (14.29) | 0 (0.00) | 5 (14.29) |  |
| **Echogenicity** |  |  |  |  |  |  | <0.001 |
| Hypoechoic [%(n)] | 9 (9.38) | 22 (66.67) | 16 (88.89) | 2 (28.57) | 2 (100.00) | 26 (74.29) |  |
| Isoechoic [%(n)] | 20 (20.83) | 0 (0.00) | 0 (0.00) | 0 (0.00) | 0 (0.00) | 6 (17.14) |  |
| Hyperechoic [%(n)] | 67 (69.79) | 3 (9.09) | 0 (0.00) | 5 (71.43) | 0 (0.00) | 3 (8.57) |  |
| Mixed-echogenicity [%(n)] | 0 (0.00) | 8 (24.24) | 2 (11.11) | 0 (0.00) | 0 (0.00) | 0 (0.00) |  |
| **Adler grade** |  |  |  |  |  |  | <0.001 |
| 0 [%(n)] | 52 (54.17) | 0 (0.00) | 13 (72.22) | 3 (42.86) | 1 (50.00) | 12 (34.29) |  |
| 1 [%(n)] | 44 (45.83) | 1 (3.03) | 3 (16.67) | 3 (42.86) | 1 (50.00) | 11 (31.43) |  |
| 2 [%(n)] | 0 (0.00) | 9 (27.27) | 1 (5.56) | 0 (0.00) | 0 (0.00) | 3 (8.57) |  |
| 3 [%(n)] | 0 (0.00) | 23 (69.70) | 1 (5.56) | 1 (14.29) | 0 (0.00) | 9 (25.71) |  |
| **Homogeneity [%(n)]** | 28 (29.17) | 14 (42.42) | 8 (44.44) | 1 (14.29) | 2 (100.00) | 23 (65.71) | 0.002 |
| **Regular [%(n)]** | 96 (100.00) | 33 (100.00) | 18 (100.00) | 7 (100.00) | 2 (100.00) | 35 (100.00) | 1.000 |
| **Circumscribed [%(n)]** | 74 (77.08) | 9 (27.27) | 14 (77.78) | 0 (0.00) | 0 (0.00) | 18 (51.43) | <0.001 |
| **Linear echogenic strands [%(n)]** | 96 (100.00) | 1 (3.03) | 0 (0.00) | 6 (85.71) | 0 (0.00) | 13 (37.14) | <0.001 |
| **Anechoic area and calcification [%(n)]** | 0 (0.00) | 4 (12.12) | 8 (44.44) | 0 (0.00) | 0 (0.00) | 7 (20.00) | <0.001 |
| **Pain [%(n)]** | 0 (0.00) | 1 (3.03) | 0 (0.00) | 0 (0.00) | 0 (0.00) | 0 (0.00) | 0.439 |

Vascular lesions include hemangioma and glomus tumor; cystic lesions include sebaceous cyst and epidermoid cyst; fibrous lesions include elastofibroma, palmar fibromatosis, and fibromatous hyperplasia; peripheral nerve lesions include schwannoma and Morton’s neuroma. Other benign lesions included fat herniation, pyogenic granuloma, intramuscular myxoma, pilomatricoma, benign myeloma-like lesions, parotid adenoma, tenosynovial giant cell tumor, hematoma-related lesions, synovial chondromatosis, synovial osteochondroma, xanthoma, fat pad, and other rare benign diagnoses.

Supplement table 4. Subgroup analysis of U-net-ResNet50.

| **Stratification** | **Subgroup** | **N** | **AUC (95% CI)** | **Sensitivity % (95% CI)** | **Specificity % (95% CI)** | **Accuracy % (95% CI)** |
| --- | --- | --- | --- | --- | --- | --- |
| Size | <=5 cm | 45 | 1.000 (1.000–1.000) | 100.0 (56.6–100.0) | 97.5 (87.1–99.6) | 97.8 (88.4–99.6) |
| Size | >5 to <=10 cm | 35 | 0.931 (0.793–1.000) | 91.7 (64.6–98.5) | 95.7 (79.0–99.2) | 94.3 (81.4–98.4) |
| Size | >10 cm | 15 | 1.000 (1.000–1.000) | 100.0 (67.6–100.0) | 100.0 (64.6–100.0) | 100.0 (79.6–100.0) |
| Location | Upper limb | 28 | 1.000 (1.000–1.000) | 100.0 (56.6–100.0) | 100.0 (85.7–100.0) | 100.0 (87.9–100.0) |
| Location | Lower limb | 56 | 0.958 (0.866–1.000) | 94.1 (73.0–99.0) | 97.4 (86.8–99.5) | 96.4 (87.9–99.0) |
| Location | Trunk | 11 | 0.917 (0.667–1.000) | 100.0 (43.9–100.0) | 87.5 (52.9–97.8) | 90.9 (62.3–98.4) |
| Pathology | Dedifferentiated liposarcoma vs all benign | 80 | 0.996 (0.983–1.000) | 100.0 (72.2–100.0) | 97.1 (90.2–99.2) | 97.5 (91.3–99.3) |
| Pathology | Myxoid liposarcoma vs all benign | 80 | 0.933 (0.796–1.000) | 90.0 (59.6–98.2) | 97.1 (90.2–99.2) | 96.3 (89.5–98.7) |
| Pathology | Pleomorphic liposarcoma vs all benign | 75 | 0.994 (0.974–1.000) | 100.0 (56.6–100.0) | 97.1 (90.2–99.2) | 97.3 (90.8–99.3) |
| Pathology | All liposarcomas vs Lipoma | 55 | 0.963 (0.893–1.000) | 96.0 (80.5–99.3) | 96.7 (83.3–99.4) | 96.4 (87.7–99.0) |
| Pathology | All liposarcomas vs Schwannoma | 43 | 0.969 (0.900–1.000) | 96.0 (80.5–99.3) | 94.4 (74.2–99.0) | 95.3 (84.5–98.7) |
| Pathology | All liposarcomas vs Fibroma | 37 | 0.983 (0.940–1.000) | 96.0 (80.5–99.3) | 100.0 (75.8–100.0) | 97.3 (86.2–99.5) |
| Pathology | All liposarcomas vs Other benign masses | 35 | 0.980 (0.928–1.000) | 96.0 (80.5–99.3) | 100.0 (72.2–100.0) | 97.1 (85.5–99.5) |
